# Clinical Outcomes of Insulin Glargine U300 on Glycemic Control and Hypoglycemia: A Retrospective Observational Study

**DOI:** 10.3390/jcm15093529

**Published:** 2026-05-05

**Authors:** Gökçen Güngör Semiz, Mehmet Çağrı Ünal, İsmail Selimoğlu, Sıla Kalender, Ege Erbay, Mehmet Emin Arayici, Abdurrahman Çömlekçi, Serkan Yener, Tevfik Demir

**Affiliations:** 1 Division of Endocrinology and Metabolism, Department of Internal Medicine, Faculty of Medicine, Dokuz Eylul University, 35330 İzmir, Türkiye; munalcagri@gmail.com (M.Ç.Ü.);; 2 Department of Internal Medicine, Faculty of Medicine, Dokuz Eylul University, 35330 Izmir, Türkiye; 3 Department of Biostatistics and Medical Informatics Faculty of Medicine, Dokuz Eylül University, 35340 İzmir, Türkiye; mehmet.e.arayici@gmail.com; 4 Department of Public Health, Faculty of Medicine, Dokuz Eylül University, 35330 İzmir, Türkiye

**Keywords:** basal insulin analogs, diabetes mellitus, metabolic control, oral antidiabetic agents, real-world evidence

## Abstract

**Background/Objectives:** Insulin glargine U300 (IGlarU300) is a second-generation, long-acting insulin analog designed to provide a more stable pharmacokinetic profile compared to insulin glargine U100. However, long-term real-world data reflecting its long-term impact on glycemic control and hypoglycemia across diverse populations remain limited. This study evaluated the 24-month clinical outcomes of transitioning to IGlarU300 in a real-world setting. **Methods:** This retrospective, single-center, observational study enrolled patients with type 1 (T1DM) or type 2 diabetes mellitus (T2DM) who transitioned to IGlarU300 between 2017 and 2021. HbA1c levels, body weight, insulin doses, and hypoglycemia rates were evaluated at baseline and up to 24 months. **Results:** A total of 242 patients (T1DM: *n* = 68, T2DM: *n* = 174) were analyzed. HbA1c levels significantly declined at all follow-up points compared to baseline (mean change at 12 months: −0.85% [95% CI: −1.24 to −0.47%]; *p* < 0.001]). No significant change in total insulin dose was observed over the one-year follow-up; however, improved glycemic control led to a significant reduction in oral antidiabetic medication use, reflecting successful treatment simplification and a decrease in polypharmacy burden (mean change: -0.50 [95% CI: −0.70 to −0.30]; *p* < 0.001). Notably, both severe and mild hypoglycemia episodes showed significant reductions (*p* = 0.010 and *p* = 0.019, respectively). Switching to IGlarU300 was associated with sustained improvements in glycemic control and a reduction in hypoglycemia rates. **Conclusions:** These findings suggest that IGlarU300 may be an effective clinical option for optimizing metabolic outcomes, though further controlled studies are warranted to confirm these observational results.

## 1. Introduction

Diabetes mellitus remains one of the most pressing global health challenges of the 21st century. According to recent International Diabetes Federation (IDF) data, approximately 589 million adults are currently living with diabetes worldwide, a figure projected to rise to 853 million by 2050 [1]. This escalating prevalence underscores the urgent need for effective management strategies to mitigate the long-term socioeconomic and clinical burdens of the disease.

Diabetes is recognized as the most critical risk factor for cardiovascular disease (CVD), contributing significantly to increased morbidity and mortality. Beyond established diabetic populations, emerging evidence suggests that glycated hemoglobin (HbA1c) levels correlate with parameters of cardiovascular dysfunction even in individuals without diabetes. Recent studies have highlighted that elevated HbA1c, even within the non-diabetic range, is associated with subclinical cardiac impairment, reinforcing the importance of precise glycemic monitoring across the metabolic spectrum [2].

Insulin therapy remains a cornerstone for T1DM and T2DM management. In patients with T2DM, treatment intensification with basal insulin is a recommended strategy, though it may be influenced by various clinical considerations [3,4]. While factors such as regimen complexity and concerns over hypoglycemia or weight gain were historically viewed as major barriers [5,6,7,8], advancements in 21st-century insulin therapy have significantly mitigated these challenges. Conventional basal regimens sometimes required higher doses or more frequent injections to reach targets, potentially increasing the risk of ‘insulin stacking’—the cumulative effect of overlapping doses—and subsequent ‘defensive eating’ [9,10]. However, the development of modern insulin analogs has enhanced the safety and efficacy profile of therapy. Nevertheless, striking a balance between achieving optimal HbA1c levels to prevent complications and minimizing the risk of hypoglycemia remains a fundamental principle of effective diabetes management [11].

To address these challenges, second-generation basal insulin analogs, such as insulin glargine U300 (IGlarU300), were developed. IGlarU300 provides a more stable and prolonged pharmacokinetic profile compared to its predecessors, offering a flatter action profile and reduced glycemic variability [12,13]. While randomized controlled trials (RCTs) have established their efficacy, there is a scientific gap regarding their performance in diverse, unselected populations over the long term. This study aims to evaluate the 24-month real-world clinical outcomes of patients transitioning to IGlarU300. We hypothesize that this transition will lead to sustained glycemic improvement and reduced hypoglycemia rates while maintaining weight stability in routine clinical practice.

## 2. Materials and Methods

### 2.1. Study Design

This single-center, retrospective, longitudinal, observational study examined patients who visited our hospital’s endocrinology outpatient clinic from March 2017 to June 2021, diagnosed with type 1 (T1DM) or type 2 diabetes mellitus (T2DM). The study protocol was approved by the Non-Interventional Research Ethics Committee of Dokuz Eylul University (decision number 2022/07-17, dated 23 February 2022).

The inclusion criteria were as follows: patients aged 18 years or older, with a diagnosis of T1DM or T2DM, who were receiving either insulin therapy (basal, basal-bolus, or premixed) or oral antidiabetic agents, and who switched to insulin glargine U300 treatment due to suboptimal glycemic control and a high frequency of hypoglycemic episodes. ‘Suboptimal glycemic control’ was defined as an HbA1c level > 7.5% despite ongoing insulin therapy. ‘High frequency of hypoglycemia’ referred to two or more episodes of mild hypoglycemia per week, or at least one episode of severe hypoglycemia within the previous three months. The exclusion criteria were as follows: individuals who transitioned to any insulin regimen other than insulin glargine U300; patients who were hospitalized due to acute coronary syndrome, acute cerebrovascular events, or acute heart failure at the time of treatment initiation; those with chronic liver disease, or cancer; pregnant woman, patients undergoing glucocorticoid treatment for any condition; and those grappling with alcohol or substance use disorders.

To minimize selection and information biases, we utilized consecutive sampling and independent double data extraction from electronic health records. A total of 242 consecutive patients were enrolled; as no a priori sample size was calculated due to the study’s retrospective nature, post hoc analysis confirmed >90% power to detect significant changes in HbA1c. Potential confounding was addressed by adjusting for baseline covariates, including age, sex, BMI, diabetes duration, diabetes type, and baseline HbA1c. All extracted data were cross-verified, and missing values were managed through complete-case analysis to ensure the integrity of the 24-month longitudinal data. Consequently, only patients with a full set of data at all major time points (Baseline, 6, 12, and 24 months) were included in the final longitudinal evaluations.

### 2.2. Data Collection

Patient records were retrospectively reviewed using the hospital’s digital database. Collected data included demographics, body mass index, comorbid conditions and complications, current treatments (number of oral antidiabetic agents, total insulin dose, and number of daily injections), frequency of hypoglycemic episodes, and laboratory parameters (such as HbA1c, fasting blood glucose, low-density lipoprotein [LDL], triglycerides, and spot urine microalbumin). Following the switch to IGlarU300, laboratory results, hypoglycemia frequency, and changes in treatment (number of oral antidiabetic agents, insulin dose, and number of injections) were evaluated at 3, 6, 12, and 24 months.

Hypoglycemia is defined by the American Diabetes Association (ADA) as a blood glucose level of 70 mg/dL or lower, often accompanied by specific symptoms. Mild hypoglycemia was classified as episodes that could be managed without assistance, while severe hypoglycemia was defined as events requiring help or medical intervention [14]. All patients received structured education on hypoglycemia from diabetes nurse educators, and hypoglycemic episodes were recorded by patient self-report during routine outpatient visits covering the preceding one-month period.

### 2.3. Treatment Schedule

Patients received individualized medical nutrition therapy as part of standard clinical care, following the guidelines of ADA and the Turkish Society of Endocrinology and Metabolism (TEMD) guidelines [14,15]. Glycemic targets were set at 80–130 mg/dL for fasting plasma glucose (FPG) and <160 mg/dL for postprandial glucose according to TEMD guidelines. Transition to IGlarU300 involved initiating therapy at the previous dose or adjusting by ±20% based on hypoglycemia history or baseline glycemic control. Dose titrations were performed every 7–10 days using seven-point self-monitoring of blood glucose (SMBG). Patients were instructed to perform measurements pre-prandially and two hours post-prandially for breakfast, lunch, and dinner, with an additional measurement at 23:00. Furthermore, to specifically monitor for nocturnal hypoglycemia, patients were required to perform a supplemental blood glucose check at 03:00 once weekly. İnsulın doses were increased by 2–8 units if FPG exceeded 130 mg/dL and reduced by 2–8 units if FPG fell below 80 mg/dL. Insulin dose adjustments were conducted systematically in accordance with TEMD’s clinical practice guidelines. Therapeutic strategies were tailored by diabetes type: T1DM management focused on systematic insulin titration, whereas T2DM centered on treatment optimization. In T2DM patients above target, OADs were adjusted per ADA/EASD and TEMD guidelines to leverage insulin-sparing effects and simplify regimens.

### 2.4. Statistical Analysis

Descriptive statistics, encompassing frequencies, percentages, means, and standard deviations, were meticulously calculated for the patient data. To thoughtfully evaluate the normality of the data distribution, we employed the Shapiro–Wilk and Kolmogorov–Smirnov tests. To assess the differences between variables, we tailored our approach based on their distribution. For normally distributed variables, we used the paired sample *t*-test. For categorical variables, we analyzed the data using the chi-squared test and, when appropriate, Fisher’s exact test. Within-subgroup change in HbA1c (12 months − baseline) was evaluated by paired-samples *t*-test, reported as mean difference (Δ) with 95% confidence interval (CI) and standardized effect size (Cohen’s d for paired data). Between-subgroup differences in ΔHbA1c were tested by Welch’s independent-samples t-test on the within-subject change scores (p-interaction). Independent predictors of ΔHbA1c were identified by multivariable linear regression adjusting for age, sex, BMI, diabetes duration, diabetes type, and baseline HbA1c. All analyses used complete paired HbA1c data (*n* = 117 with baseline and 12-month values; *n* = 62 with complete data for all regression covariates). All statistical analyses were carried out using the Statistical Package for the Social Sciences (SPSS), version 30.0. We defined statistical significance as a *p*-value < 0.05, ensuring robust evaluation of our findings.

## 3. Results

### 3.1. Baseline Characteristics

A total of 242 patients with diabetes mellitus (DM) were enrolled in this study, including 139 females and 103 males, with a mean age of 55.47 ± 15.88 (18–88) years. Of these patients, 68 had type 1 diabetes mellitus (T1DM), and 174 had type 2 diabetes mellitus (T2DM). The baseline treatment characteristics are comprehensively presented in Table 1. At study entry, the vast majority of the cohort (*n* = 238, 98.4%) was already receiving insulin therapy, including basal insulin alone, basal-plus regimens, or intensive basal-bolus therapy, often in combination with OADs. A small subgroup of four patients (1.6%) transitioned to IGlarU300 from OAD monotherapy as a treatment intensification strategy.

Hypertension was the most common comorbidity, followed by hyperlipidemia, coronary artery disease, and chronic renal failure. Regarding current treatment regimens, 47.1% of patients received a combination of oral antidiabetic drugs (OADs) and intensive insulin therapy, 32.2% received intensive insulin therapy alone, and 17.4% received OADs plus basal insulin. The most commonly used OADs were metformin, dipeptidyl peptidase-4 (DPP-4) inhibitors, and sodium-glucose cotransporter-2 (SGLT2) inhibitors, in that order. Demographic characteristics of the study population are presented in Table 1.

### 3.2. Primary Glycemic Outcomes and Longitudinal HbA1c Response

A total of 39 patients discontinued treatment during follow-up. The reasons for discontinuing IGlarU300 are detailed in Table 2. Notably, there was no significant difference in discontinuation rates between the Type 1 Diabetes Mellitus (T1DM) and Type 2 Diabetes Mellitus (T2DM) groups (*p* = 0.871). Approximately 21.1% of patients with T1DM and 22.1% of those with T2DM opted to halt their treatment regimen, with poor metabolic control emerging as the most prevalent reason for this decision.

**Figure 1:**
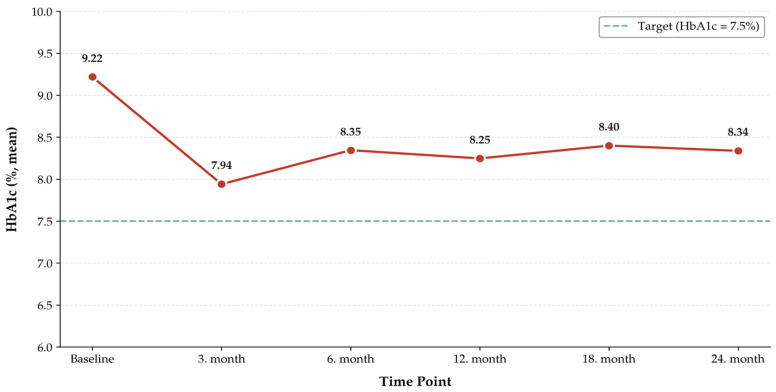
Change in HbA1c after treatment change.

When comparing glycemic control at 3, 6, 12, and 24 months after IGlarU300 initiation to baseline, HbA1c decreased at each time point. Baseline HbA1c was 9.21%, which declined to 7.94% at 3 months, 8.34% at 6 months, 8.24% at 12 months, and 8.33% at 24 months. The treatment efficacy was significant at months 3, 6, and 12 (*p* < 0.001), with sustained results through month 24 (*p* = 0.002) (Figure 1).

**Figure 2:**
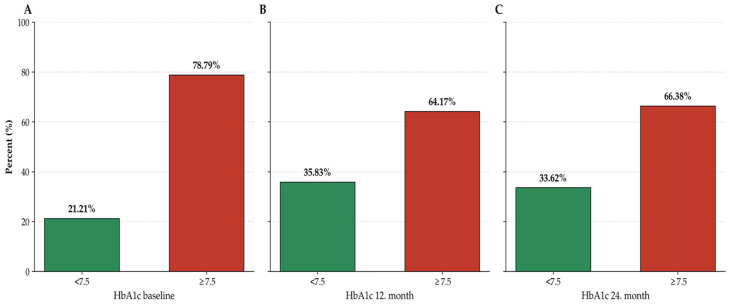
(**A**) Proportion of patients with Hba1c < 7.5 and below at baseline. (**B**) Proportion of patients with Hba1c < 7.5 at 12 months after treatment change (**C**) Proportion of patients with HbA1c < 7.5 at 24 months after treatment change.

One year after transitioning to IGlarU300, the proportion of patients with HbA1c levels ≤ 7.5% increased from 21.21% to 35.83%. By the end of the second year, this rate was 33.62% (Figure 2).

Additionally, a significant reduction in HbA1c was observed in both T1DM (Mean change: −0.54%; 95% CI: [−1.02, −0.05]; *p* = 0.029) and T2DM (Mean change: −1.04%; 95% CI: [−1.58, −0.50]; *p* < 0.001) subgroups after 12 months.

There were no significant changes in LDL and triglyceride levels over 12 months (*p* = 0.973, *p* = 0.132, respectively). Similarly, the mean body weight remained stable throughout the study period; mean BMI was 28.52 ± 6.64 kg/m^2^ at baseline and 28.51 ± 5.89 kg/m^2^ at 12 months (*p* = 0.982). Changes in patients’ metabolic status over time are presented in Table 3.

Alterations in the treatment regimen were evaluated throughout follow-up. The transition to IGlarU300 was characterized by dose neutrality. No significant change in total daily insulin dose was observed at the time of switching (*p* = 0.200). Furthermore, longitudinal analysis demonstrated that neither the IGlarU300 dose (*p* = 0.945) nor total daily insulin requirements (*p* = 0.489) showed a significant increase over the 12-month follow-up period. In addition, among patients with T2DM, the number of oral antidiabetic agents used significantly decreased over the 12-month period (*p* < 0.001). A comparison of patients’ initial treatment regimens and those after transitioning to IGlarU300 is presented in Table 4.

### 3.3. Evaluation of Hypoglycaemia

The frequency of mild hypoglycemic events decreased from 1 (0.0; 3.0) events per month to 1 (0.0; 2.0) events per month (*p* = 0.010) following the initiation of IGlarU30.0 therapy. There was also a significant reduction in the frequency of severe hypoglycemic events, from 0 (0.0; 0.0) events per month to 0 (0.0; 0.0) events per month (*p* = 0.019). A significant decrease in mild hypoglycemic events was observed in both T1DM (Mean change: −1.47; 95% CI: [−2.77, −0.18]; *p* = 0.026) and T2DM (Mean change: −1.92; 95% CI: [−3.82, −0.02]; *p* = 0.047) subgroups. Data regarding the frequency and severity of hypoglycemic events are presented in Table 4.

### 3.4. Subgroup Analysis of Glycemic Efficacy

Overall, HbA1c fell by −0.85 percentage points at 12 months (95% CI −1.24 to −0.47, *p* < 0.001; paired *n* = 117), representing a statistically significant and clinically meaningful improvement. Male patients experienced a substantially larger reduction (Δ = −1.42%, 95% CI −2.06 to −0.78) than female patients (Δ = −0.52%, 95% CI −0.99 to −0.05); the between-sex difference was statistically significant (p-interaction = 0.025). This was partly explained by higher baseline HbA1c in men (9.66 vs 8.78). Both age strata improved significantly; the reduction was numerically larger in patients aged ≥55 (Δ = −1.08%) than in those <55 (Δ = −0.59%), but the between-group difference did not reach statistical significance (p-interaction = 0.205). Non-obese patients (BMI < 30) showed a modest reduction (Δ = −0.58%, *p* = 0.086), whereas obese patients (BMI ≥ 30) showed essentially no change at 12 months (Δ = −0.14%, *p* = 0.708). The between-group difference was not statistically significant (p-interaction = 0.381), which may reflect the limited BMI-stratified sample size (*n* = 64 with complete BMI data). Diabetes duration: Patients with shorter disease duration (<10 years) experienced a markedly larger HbA1c reduction (Δ = −1.46%, 95% CI −2.49 to −0.43) than those with longer duration (≥10 years; Δ = −0.56%, 95% CI −0.95 to −0.18); the between-group difference approached but did not reach statistical significance (p-interaction = 0.106). The results of the subgroup analyses are presented in Table 5.

### 3.5. Multivariable Predictors of Treatment Response

Multivariable model: After simultaneous adjustment for age, sex, BMI, diabetes duration, diabetes type, and baseline HbA1c, four predictors were independently associated with ΔHbA1c (model R^2^ = 0.449, *p* < 0.001): (i) higher baseline HbA1c (β = −0.623 per 1%, *p* < 0.001) and older age (β = −0.040 per year, *p* = 0.022) predicted greater HbA1c reduction, whereas (ii) longer diabetes duration (β = +0.070 per year, *p* = 0.001) and higher BMI (β = +0.065 per kg/m^2^, *p* = 0.048) were independently associated with smaller HbA1c reduction. Diabetes type was not an independent predictor after adjustment (Table 6).

## 4. Discussion

Insulin therapy remains the cornerstone of glycemic management across the spectrum of diabetes. While it is an indispensable life-sustaining replacement therapy for Type 1 Diabetes Mellitus (T1DM), it also plays a critical role in managing unstable or advanced Type 2 Diabetes Mellitus (T2DM) [16]. The long-term benefits of early intensive glycemic control have been further solidified by the 44-year follow-up of the UKPDS 91 study, which demonstrates a sustained ‘legacy effect’ on mortality and complications in T2DM [17]. Similarly, achieving stable glycemic targets in T1DM is paramount to preventing both acute metabolic crises and chronic microvascular damage. Furthermore, the ADA 2026 Standards of Care continue to support the early initiation of insulin in T2DM patients with significant glucose toxicity or high baseline HbA1c to achieve rapid metabolic stabilization [18]. In this context, second-generation long-acting insulin analogs like IGlarU300 offer a more stable, peakless, and prolonged pharmacokinetic profile than Gla-100, providing consistent glucose regulation for up to 36 h [19]. These pharmacological advantages are particularly beneficial for both T1DM and T2DM patients by reducing glycemic variability and the risk of nocturnal hypoglycemia.

Our findings demonstrate the clinical utility of IGlarU300’s stable profile in a real-world setting. Despite a high baseline HbA1c (9.21%), significant and sustained improvements were achieved at 12 and 24 months (8.24% and 8.33%, respectively). A key clinical implication is the simplification of treatment regimens; the average number of concomitant OADs decreased significantly (1.39 to 0.88; *p* < 0.001) without necessitating an increase in total insulin dose (59.06 IU vs. 61.37 IU, *p* = 0.200). The observed reduction in the polypharmacy burden, alongside the increase in the proportion of patients reaching HbA1c targets ≤ 7.5% (from 19.5% to 38.9%), suggests that IGlar U300 may facilitate a more favorable titration profile. This could allow for intensive glycemic management with a reduced risk of hypoglycemic events in a real-world clinical setting. This dual clinical benefit—attaining glycemic targets while maintaining patient safety—is consistent with the favorable stability and pharmacokinetic profile of IGlar U300 compared to earlier-generation basal insulins. Regarding the predictors of clinical response, our multivariable analysis (*n* = 62) provided exploratory insights. Higher baseline HbA1c and older age (β = −0.040, *p* = 0.022) were significantly associated with greater HbA1c reduction. The enhanced response in the elderly underscores the advantage of a peakless pharmacokinetic profile, which enables more confident titration in a population traditionally vulnerable to hypoglycemia. Conversely, longer diabetes duration and higher BMI were associated with a more modest response, likely reflecting advanced beta-cell exhaustion and underlying insulin resistance. Notably, observed sex-based differences were primarily driven by higher baseline levels in the male cohort rather than by biological sex alone.

Our results align with the Evolution [20] and Turkish Ease [21] studies, which similarly reported significant reductions in HbA1c and target attainment in real-world cohorts. Furthermore, the decrease in hypoglycemic frequency observed in our study is consistent with large-scale meta-analytic evidence [22] supporting IGlarU300’s favorable safety profile relative to first-generation analogs. A notable point of divergence from studies in the Gulf [23] and India [24]—where glycemic control required significant insulin dose escalations—is that our cohort maintained stable doses throughout the follow-up. Crucially, this stability enabled the simplification of complex polypharmacy regimens without compromising long-term metabolic control, suggesting that the transition to IGlarU300 provides potent yet manageable basal coverage in routine clinical practice.

Our study is subject to several limitations that warrant careful consideration when interpreting the findings. First, the retrospective design relies on electronic health records, which introduces potential information bias and results in missing data at various follow-up intervals. For instance, while baseline HbA1c data were available for 231 patients, primary outcome measures such as BMI were available for only 86 patients at the 12-month follow-up. Second, the absence of an active control group (e.g., patients continuing on first-generation basal analogs) limits our ability to definitively isolate the therapeutic effect of IGlarU300 from the general impact of standard clinical care or “study effect”. Third, as a single-center study conducted at a tertiary care hospital, our cohort likely represents a more complex patient population, which may limit the generalizability of these results to the broader diabetic community in primary care settings. Also, the assessment of hypoglycemia was based on patient self-reports, which may be subject to recall bias or underreporting. Although patients were encouraged to maintain accurate logs, the lack of systematic laboratory or glucose sensor confirmation for every reported event may influence the estimated frequency of hypoglycemic episodes. Finally, although the total sample size was robust (=242), not all patients completed the full 24-month follow-up, which may have influenced the long-term statistical power for certain subgroups. Potential confounders, such as changes in dietary habits, physical activity levels, and medication adherence, were not strictly controlled or quantified. While we adjusted for baseline clinical characteristics (age, sex, BMI, diabetes duration, diabetes type, and baseline HbA1c), these unmeasured lifestyle factors may have influenced the observed glycemic outcomes. Despite these constraints, the primary strength of this study is its real-world design, which reflects the long-term clinical outcomes of IGlarU300 in an unselected, diverse patient population, rather than the highly controlled environment of randomized trials.

## 5. Conclusions

In summary, our 24-month real-world study indicates that transitioning to IGlarU300 is associated with a sustained, clinically significant reduction in HbA1c, while maintaining a favorable safety profile with reduced incidences of both mild and severe hypoglycemia. A noteworthy observation is the significant reduction in the oral antidiabetic (OAD) medication burden, suggesting that IGlarU300 may facilitate the simplification of complex treatment regimens without requiring an increase in total insulin dosage. Furthermore, our multivariable analysis provides exploratory insights into the clinical application of these findings, identifying higher baseline HbA1c and older age as independent predictors of a more pronounced glycemic response, whereas obesity and longer disease duration appear to be key barriers to optimal outcomes. Overall, these findings suggest that IGlarU300 remains a sustainable and well-tolerated basal insulin option in routine practice. Our data indicate that the efficacy observed in randomized controlled trials is reflected within the complexities of daily clinical care, supporting its utility in long-term diabetes management.

## Figures and Tables

**Table 1:** Characteristics of the patients before transition to IGlarU300.

Variables	
Age, years	55.47 ± 15.85
Sex	
Female	139 (57.4)
Male	103 (42.6)
Type of diabetes	
Type 1	68 (28.1)
Type 2	174 (71.9)
Duration of diabetes (months)	178 ± 115.08
Weight (kg)	81.44 ± 20.01
Co-morbidities	
HT	137 (56.6)
HL	128 (52.9)
CAD	63 (26)
PAD	16 (6.6)
CKD	43 (17.8)
CVD	14 (5.8)
CA	16 (6.6)
Microvascular complications	
Retinopathy	74 (30.6)
Nephropath	56 (23.1)
Neuropathy	77 (31.8)
Prior therapy	
Only OAD	4 (1.7)
Basal insulin	4 (1.7)
Basal insulin + OAD	42(17.4)
Intensive insulin	78 (32.2)
Intensive insulin + OAD	114(47.1)
OAD number	1.39 ± 1.27
OAD	
Metformin	150 (62)
Sulfonylureas	26 (10.7)
Pioglitazon	20 (8.3)
DPP-4 inh.	86 (35.5)
SGLT-2 inh.	52(21.5)
GLP-1 A	16 (6.6)

Data are shown as *n* (%) and given as mean ± SD. SD, standard deviation; HT, hypertension; HL, hyperlipidemia; CAD, coronary artery disease; PAD, peripheral arterial disease; CKD, chronic kidney disease; CVD, cerebrovascular disease; CA, cancer; OAD, oral antidiabetic drug; DPP-4 inh, dipeptidyl peptidase-4 inhibitor; SGLT-2 inh, sodium-glucose cotransporters-2 inhibitor; GLP-1A, Glucagon-Like Peptide-1 Analogs.

**Table 2:** Reasons for discontinuing IGlarU300 treatment.

Type of Diabetes	Number of Treatment Drop-Outs *
Type 1	12 (21.1)
Type 2	27 (22.1)
Reasons	
Poor metabolic control	14 (35.9)
Hypoglycaemia	3 (7.7)
Patient non-compliance	9 (23.1)
Pregnancy	2 (5.1)
The need for a referral to a tertiary care hospital	4 (10.3)
Mortality	5 (12.8)
Unknown	2 (5.1)
Total	39 (100)

Data are shown as *n* (%). * There was no significant difference in discontinuation rates between type 1 and type 2 diabetics (*p* = 0.871).

**Table 3:** Changes in BMI and Glycemic Control Following the Transition to IGlarU300.

Parameter	*n*	Mean ± SD		Mean Change (Δ)	95% CI (Lower–Upper)	*p*-Value *
HbA1c (%) Baseline	231	9.21 ± 2.14		N/A	N/A	N/A
HbA1c (%) (3. month)	67	7.94 ± 1.27		−1.12	[−1.54, −0.69]	<0.001
HbA1c (%) (6. month)	118	8.34 ± 1.62		−0.66	[−1.04, −0.28]	<0.001
HbA1c (%) (12. month)	117	8.24 ± 1.65		−0.85	[−1.24, −0.47]	<0.001
HbA1c (%) (24. month)	113	8.33 ± 1.80		−0.58	[−0.94, −0.21]	0.002
	*n*	Baseline (Mean ± SD)	Follow up ᵃ (Mean ± SD)	Mean Change (Δ)	95% CI (Lower–Upper)	*p*-value *
LDL levels (mg/dL)	111	119.41 ± 37.45	119.29 ± 43.46	−0.117	[−6.88, 6.65]	0.973
TG levels (mg/dL)	110	192.45 ± 163.117	170.83 ± 106.633	−21.62	[−49.85, 6.59]	0.132
BMI (kg/m^2^)	86	28.52 ± 6.64	28.51 ± 5.89	−0.006	[−0.61, 0.60]	0.982

HbA1c, glycated hemoglobin; LDL, low-density lipoprotein; TG, triglycerides; BMI, body mass index; SD, standard deviation. N/A: Not Applicable. * Paired samples T test. ᵃ 12. Monthly data.

**Table 4:** Comparison of Treatment Regimens and Hypoglycemia Incidence Before and After the Transition to IGlarU300.

	Baseline (Mean ± SD)	After Switch (Mean ± SD)	Mean Change (Δ)	95% CI (Lower–Upper)	*p*-Value *
Total İnsulın dose (IU) (*n* = 195)	59.06 ± 39.65	61.37 ± 36.57	2.31	[−1.23, 5.86]	0.200
IglarU300 dose (IU) (*n* = 132)	28.86 ± 14.91	29.36 ± 14.79 (12. month)	0.50	[−0.93, 1.95]	0.489
Total insülin dose (IU) (*n* = 125)	57.14 ± 31.35	57.05 ± 31.35 (12. month)	−0.09	[−2.84, 2.65]	0.945
Oad number (*n*) (*n* = 137)	1.39 ± 1.27	0.88 ± 1.04 (12. month)	−0.50	[−0.70, −0.30]	<0.001
Mild hypoglycaemia/month (*n* = 152)	3.39 ± 8.85	1.61 ± 3.38 (12. month)	−1.78	[−3.14, −0.42]	0.010
Severe hypoglycaemia (per month) (*n* = 152)	0.39 ± 1.46	0.10 ± 0.47 (12. month)	−0.28	[−0.53, −0.04]	0.019
Hospital admission for hypoglycaemia/month (*n* = 151)	0.13 ± 0.59	0.08 ± 0.40 (12. month)	−0.05	[−0.15, 0.04]	0.303

SD, standard deviation; IU, International Unit. * Paired samples T test.

**Table 5:** Subgroup analysis of HbA1c change from baseline to 12 months.

Subgroup	Stratum	*n*	Baseline Mean ± SD	12-Month Mean ± SD	Mean Δ	95% CI of Δ	*p* (Within)	p-İnteraction
Sex	Female	73	8.78 ± 1.74	8.26 ± 1.53	−0.52	−0.99 to −0.05	0.031	0.025
	Male	44	9.66 ± 2.21	8.24 ± 1.90	−1.42	−2.06 to −0.78	<0.001	
Age	<55	52	9.02 ± 1.86	8.44 ± 1.93	−0.59	−1.14 to −0.04	0.037	0.205
	≥55	65	9.18 ± 2.07	8.10 ± 1.44	−1.08	−1.61 to −0.54	<0.001	
BMI (kg/m^2^)	<30	37	8.73 ± 1.60	8.15 ± 1.39	−0.58	−1.24 to 0.09	0.086	0.381
	≥30	27	9.46 ± 2.14	9.32 ± 2.40	−0.14	−0.91 to 0.62	0.708	
Diabetes duration	<10 yr	32	9.26 ± 2.33	7.81 ± 1.41	−1.46	−2.49 to −0.43	0.007	0.106
	≥10 yr	68	9.04 ± 1.83	8.47 ± 1.65	−0.56	−0.95 to −0.18	0.004	

Δ = mean change from baseline to 12 months (negative values indicate reduction). Within-subgroup p from paired-samples *t*-test. p-interaction from Welch’s independent-samples *t*-test comparing ΔHbA1c between the two strata. Age cut-off based on the cohort mean (55 years); diabetes-duration cut-off set at 120 months (10 years); BMI cut-off at 30 kg/m^2^ (obesity threshold).

**Table 6:** Multivariable linear regression: independent predictors of HbA1c change at 12 months.

Predictor	β (Coefficient)	SE	95% CI	*p*-Value
Age (per year)	−0.040	0.017	−0.074 to −0.006	0.022
Male sex (vs female)	−0.728	0.390	−1.510 to 0.053	0.067
BMI (per kg/m^2^)	0.065	0.032	0.001 to 0.130	0.048
Diabetes duration (per year)	0.070	0.020	0.029 to 0.110	0.001
Type 2 DM (vs. Type 1)	0.445	0.600	−0.759 to 1.648	0.462
Baseline HbA1c (per 1%)	−0.623	0.113	−0.849 to −0.397	<0.001

Dependent variable: change in HbA1c (12-month baseline, %). *n* = 62 with complete data for all covariates. Model R^2^ = 0.449, adjusted R^2^ = 0.389, overall model *p* < 0.001. Negative β indicates greater HbA1c reduction; positive β indicates smaller reduction.

## Data Availability

The datasets used and/or analyzed in this study are available upon reasonable request from the corresponding author.
